# Prognostic Significance of Nucleated RBCs in Predicting Mortality Among ST-Elevation Myocardial Infarction Patients Admitted to the ICU

**DOI:** 10.7759/cureus.45445

**Published:** 2023-09-18

**Authors:** Syeda Akefah Hashmi, Raheela Khowaja, Maria Ali, Ali R Mangi, Aamir Khowaja, Gohar Riaz, Syed Muhammad Mahad Hashmi, Ali Raza Haider, Syed Danish Afaque Hussain, Sidrah Agha

**Affiliations:** 1 Critical Care Medicine, National Institute of Cardiovascular Diseases (NICVD), Karachi, PAK; 2 Cardiology, National Institute of Cardiovascular Diseases (NICVD), Karachi, PAK; 3 Transfusion Medicine, Regional Blood Centre Karachi, Karachi, PAK; 4 Cardiac Surgery, National Institute of Cardiovascular Diseases (NICVD), Karachi, PAK; 5 Adult Cardiology, National Institute of Cardiovascular Diseases (NICVD), Karachi, PAK

**Keywords:** prognostic markers, mortality, intensive care unit, nucleated red blood cells, st-elevation myocardial infarction (stemi)

## Abstract

Background

The nucleated red blood cells (NRBCs) are a readily available hematological parameter with potential for risk stratification for mortality. Therefore, our objective was to assess the predictive significance of NRBCs for ICU mortality among ST-elevation myocardial infarction (STEMI) patients admitted to an ICU. Additionally, we aimed to compare the predictive capacity of NRBCs with that of the acute physiology and chronic health evaluation (APACHE) II score and the sequential organ failure assessment (SOFA) score.

Methodology

This descriptive cross-sectional study was conducted in the ICU of the National Institute of Cardiovascular Diseases (NICVD) in Karachi, Pakistan, from the 1st of February to the 30th of June, 2023. We included adult patients (≥18 years) diagnosed with STEMI who were subsequently admitted to the ICU. NRBCs were assessed in all patients over up to five days at 24-hour intervals, and the highest NRBC levels were used for the final analysis. Furthermore, the APACHE II score and the SOFA score were also documented. Patients were monitored throughout their ICU stay, and any adverse events or complications, such as re-intubation, bleeding necessitating transfusion, requirement for renal replacement therapy, arrhythmias, re-infarction, and mortality, were recorded.

Results

This study included 151 patients, of whom 97 (64.2%) were male, with an average age of 61.1 ± 10.7 years. Patients with positive NRBCs had higher mean SOFA scores (7.4 ± 2.9 vs. 5.4 ± 2.6; p < 0.001) and APACHE II scores (14.6 ± 6.3 vs. 12.6 ± 5.5; p = 0.037) compared to those with negative NRBCs. The culprit vessel showed greater mean stenosis (%) in patients with positive NRBCs (98.8 ± 3.0% vs. 96.8 ± 5.7%; p = 0.004). Post-procedure thrombolysis in myocardial infarction (TIMI) flow grade III was lower in patients with positive NRBCs (77.8% vs. 91.8% for positive vs. negative NRBCs, respectively). Moreover, patients with positive NRBCs experienced significantly higher mortality rates (63% vs. 8.2%; p < 0.001), a higher occurrence of arrhythmias (35.2% vs. 19.6%; p = 0.034), and an increased requirement for vasopressors/inotropic support (96.3% vs. 71.1%; p < 0.001) compared to those with negative NRBCs. NRBCs demonstrated superior discriminatory ability compared to the SOFA and APACHE II scores, with an area under the curve of 0.818 (95% CI: 0.738-0.899) for NRBCs, 0.774 (95% CI: 0.692-0.857) for SOFA, and 0.707 (95% CI: 0.613-0.801) for APACHE II. Positive NRBCs exhibited a sensitivity of 81.0% and a specificity of 81.7% in predicting ICU mortality.

Conclusion

In conclusion, positive NRBCs emerge as a robust and reliable prognostic indicator, strongly associated with an elevated risk of ICU mortality in STEMI patients. Moreover, the predictive power of positive NRBCs surpasses that of both SOFA and APACHE II scoring systems.

## Introduction

Cardiovascular disease (CVD) stands as the leading cause of global illness and mortality. The burden of CVD is escalating at an alarming pace in low- and middle-income countries (LMICs). This situation presents a significant global concern, given that LMICs comprise roughly 80% of the world's population [1]. The prevailing and gravely serious form of CVD is known as ST-segment elevation myocardial infarction (STEMI) [2]. Though outcomes for STEMI patients have notably improved due to the widespread use of primary percutaneous coronary intervention (PCI), evidence-based pharmacological protocols, and advancements in stenting and deployment techniques [2,3], STEMI remains a leading cause of premature mortality and contributes significantly to disability-adjusted life years (DALYs) worldwide, especially in LMICs [1]. However, 2.5%-10% of patients with STEMI experience unfavorable outcomes within 30 days after the initial procedure [4-6]. Consequently, in clinical practice, it is imperative to identify patients at elevated risk of adverse events [7], giving physicians an opportunity to proactively address the potential burden of post-procedure complications [8].

Dissimilar to the majority of other eukaryotic cells, fully developed RBCs lack a nucleus. Nevertheless, when subjected to stress, the bone marrow endeavors to counteract anoxemia by releasing premature NRBCs into circulation [9]. Numerous studies have shown that an increased presence of these cells in the peripheral blood of various hospitalized patients correlates with heightened stimulus and disruption of normal barriers [10]. Such cells have been associated with negative outcomes in the ICU and during hospitalization [11,12]. Conversely, a decline in their prevalence has been linked to potential improvements in outcomes [13]. Hematological parameters, easily obtainable and monitorable on a routine basis, have demonstrated strong associations with the progression of various organ diseases, including those of the myocardium, pleura, GI tract, and sepsis [14-16]. In resource-limited healthcare settings, the burden of CVDs could potentially be alleviated by measurements capable of addressing multi-organ involvement simultaneously.

Several scoring systems have been developed to quantify the likelihood of mortality and morbidity in critically ill patients. Among them, the well-known acute physiology and chronic health evaluation (APACHE) score was introduced in 1981 [17]. Over time, four iterations have emerged, with APACHE II being the most widely adopted version. Its scoring ranges from 0 to 71, with higher scores indicating an elevated mortality risk. While primarily used in medical intensive care, APACHE II has been applied to STEMI patients in multiple studies, reaffirming its value [18,19]. Given its ease of assessment and ready availability, our objective was to assess the predictive significance of nucleated red blood cells (NRBCs) in predicting ICU mortality among STEMI patients admitted to the ICU. Additionally, we aimed to compare the predictive capacity of NRBCs with that of the APACHE II score and the sequential organ failure assessment (SOFA) score.

## Materials and methods

This descriptive cross-sectional study was conducted within the ICU of the National Institute of Cardiovascular Diseases (NICVD) in Karachi, Pakistan, spanning from the 1st of February to the 30th of June 2023. The study received approval from the institutional review board (IRB), reference number: IRB/14/2023, and verbal informed consent was obtained. The inclusion criteria stipulated adult patients (≥ 18 years) with a diagnosis of STEMI who were subsequently admitted to the ICU. However, individuals with established hematological disorders, a history of steroid usage, liver/spleen ailments, and patients presenting with identified sources of infection, positive cultures, or elevated procalcitonin levels were excluded from the study.

The diagnosis of STEMI was established by consultant cardiologists using presenting symptoms (typical chest pain for at least 20 minutes) and the baseline 12-lead ECG in accordance with the fourth universal definition of myocardial infarction [20]. The specific ECG criteria included ST elevation in at least two contiguous leads: >2mm in men or >1mm in women in leads V2 to V3 and/or >1mm in other contiguous chest or limb leads. All patients received treatment according to the standard institutional protocols. Data for this study were collected using a structured proforma, which encompassed patients' demographic and clinical characteristics, as well as their outcomes. NRBCs were assessed in all patients over a period of up to five days, with a 24-hour interval, and the highest NRBC levels were utilized for the final analysis. Furthermore, the APACHE II score and the SOFA score were also documented. Patients were monitored throughout their ICU stay, and any adverse events or complications, such as re-intubation, bleeding necessitating transfusion, the requirement for renal replacement therapy, arrhythmias, re-infarction, and mortality, were recorded. Additional data were also documented regarding the need for vasopressors/inotropic support, the use of an intra-aortic balloon pump (IABP), and the duration of ICU and hospital stays.

Statistical analysis was executed using SPSS version 21 (IBM Corp., Armonk, NY, USA). Patients were categorized into two groups: NRBC positive (NRBCs > 0) and NRBC negative (NRBCs = 0). Data were summarized using mean ± SD and frequency (%), and the two groups were compared using independent sample t-tests and Chi-square tests. Receiver operating characteristic curve (ROC) analysis was carried out for NRBCs, the APACHE II score, and the SOFA score, with ICU mortality as the discriminating variable. The corresponding area under the curve (AUC) and its 95% CI were determined. All analyses were conducted at a significance level of 5%.

## Results

This study encompassed a cohort of 151 patients, of which 97 (64.2%) were male, with a mean age of 61.1 ± 10.7 years. Nearly all patients, 148 (98%), underwent intubation, while 42 (27.8%) had undergone cardiopulmonary resuscitation in the past. During the presentation, 30 (19.9%) patients were classified as Killip class IV and 95 (62.9%) were classified as Killip class III. Positive results for NRBCs were identified in 54 (35.8%) patients (Table 1). In comparison to patients with negative NRBCs, those with positive NRBCs exhibited a significant association with elevated SOFA and APACHE II scores upon admission, displaying mean values of 7.4 ± 2.9 versus 5.4 ± 2.6 (p < 0.001) and 14.6 ± 6.3 versus 12.6 ± 5.5 (p = 0.037), respectively.

**Table 1:** Comparison of clinical and demographic data for the patients with and without positive NRBCs. NRBCs: Nucleated red blood cells; MI: Myocardial infarction; SOFA: Sequential organ failure assessment; APACHE: Acute physiology and chronic health evaluation. *based on patients with cardiopulmonary resuscitation

	Total	NRBCs		P-value
		Negative	Positive	
Total (N)	151	97 (64.2%)	54 (35.8%)	-
Gender				
Male	97 (64.2%)	65 (67%)	32 (59.3%)	0.341
Female	54 (35.8%)	32 (33%)	22 (40.7%)	
Age (years)	61.1 ± 10.7	61.9 ± 10.7	59.7 ± 10.7	0.234
Killip class				
I	13 (8.6%)	8 (8.2%)	5 (9.3%)	0.928
II	13 (8.6%)	9 (9.3%)	4 (7.4%)	
III	95 (62.9%)	62 (63.9%)	33 (61.1%)	
IV	30 (19.9%)	18 (18.6%)	12 (22.2%)	
Intubation	148 (98%)	95 (97.9%)	53 (98.1%)	0.929
Catheterization laboratory	33 (21.9%)	21 (21.6%)	12 (22.2%)	0.767
Intensive care unit	17 (11.3%)	9 (9.3%)	8 (14.8%)	
Emergency department	98 (64.9%)	65 (67%)	33 (61.1%)	
Cardiopulmonary resuscitation (CPR)	42 (27.8%)	24 (24.7%)	18 (33.3%)	0.259
*Rhythm at the time of cardiopulmonary resuscitation				
Asystole	4 (9.5%)	3 (12.5%)	1 (5.6%)	0.676
Pulseless electrical activity (PEA)	3 (7.1%)	1 (4.2%)	2 (11.1%)	
Ventricular tachycardia	20 (47.6%)	10 (41.7%)	10 (55.6%)	
Ventricular fibrillation	5 (11.9%)	3 (12.5%)	2 (11.1%)	
Bradyarrest	10 (23.8%)	7 (29.2%)	3 (16.7%)	
*Duration of cardiopulmonary resuscitation				
Less than 6 minutes	14 (33.3%)	8 (33.3%)	6 (33.3%)	0.608
6 to 10 minutes	15 (35.7%)	10 (41.7%)	5 (27.8%)	
10 to 14 minutes	5 (11.9%)	3 (12.5%)	2 (11.1%)	
More than 14 minutes	8 (19%)	3 (12.5%)	5 (27.8%)	
Type of myocardial infarction (MI)				
Anterolateral wall MI	6 (4%)	6 (6.2%)	0 (0%)	0.566
Anterior wall MI	88 (58.3%)	58 (59.8%)	30 (55.6%)	
Anterior wall MI with RBBB	13 (8.6%)	9 (9.3%)	4 (7.4%)	
Infero-posterior wall MI	10 (6.6%)	7 (7.2%)	3 (5.6%)	
Infero-posterior wall MI with RV infract	2 (1.3%)	1 (1%)	1 (1.9%)	
Inferior wall MI	14 (9.3%)	7 (7.2%)	7 (13%)	
Inferior wall MI with RV infarct	14 (9.3%)	7 (7.2%)	7 (13%)	
Lateral wall MI	2 (1.3%)	1 (1%)	1 (1.9%)	
Posterior wall MI	2 (1.3%)	1 (1%)	1 (1.9%)	
SOFA Score at admission	6.1 ± 2.9	5.4 ± 2.6	7.4 ± 2.9	<0.001
APACHE ll Score at admission	13.3 ± 5.9	12.6 ± 5.5	14.6 ± 6.3	0.037

In comparison to patients with negative NRBCs, those with positive NRBCs exhibited a significant association with higher stenosis (%) in the culprit vessel, showcasing mean values of 98.8 ± 3.0% versus 96.8 ± 5.7% (p = 0.004). Moreover, positive NRBC findings were also correlated with impaired post-procedure thrombolysis in myocardial infarction (TIMI) flow (p = 0.023), resulting in a TIMI III flow rate of 77.8% for patients with positive NRBCs in contrast to 91.8% for patients without positive NRBCs (Table 2). Likewise, when comparing patients with negative NRBCs to those with positive NRBCs, a substantial association emerged with elevated rates of mortality (63% vs. 8.2%; p < 0.001), arrhythmias (35.2% vs. 19.6%; p = 0.034), and the requirement for vasopressors/inotropic support (96.3% vs. 71.1%; p < 0.001).

**Table 2:** Comparison of angiographic data and hospital course for the patients with and without positive NRBCs. NRBCs: Nucleated red blood cells; IABP: Inter-aortic balloon pump.

	Total	NRBCs		P-value
		Negative	Positive	
Total (N)	151	97 (64.2%)	54 (35.8%)	-
Culprit vessel				
Left main	2 (1.3%)	1 (1%)	1 (1.9%)	0.473
Left anterior descending artery	106 (70.2%)	72 (74.2%)	34 (63%)	
Right coronary artery	25 (16.6%)	13 (13.4%)	12 (22.2%)	
Left circumflex	18 (11.9%)	11 (11.3%)	7 (13%)	
Number of vessels involved				
Single vessel disease	37 (24.5%)	24 (24.7%)	13 (24.1%)	0.368
Two vessel disease	46 (30.5%)	33 (34%)	13 (24.1%)	
Three vessel disease	68 (45%)	40 (41.2%)	28 (51.9%)	
Pre-procedure thrombolysis in myocardial infarction (TIMI) flow				
0	109 (72.2%)	63 (64.9%)	46 (85.2%)	0.050
I	27 (17.9%)	21 (21.6%)	6 (11.1%)	
II	11 (7.3%)	9 (9.3%)	2 (3.7%)	
III	4 (2.6%)	4 (4.1%)	0 (0%)	
Stenosis of culprit vessel (%)	97.5 ± 5	96.8 ± 5.7	98.8 ± 3.0	0.004
Total ischemia time (hours)	11.6 ± 9.8	11.5 ± 9	11.9 ± 11.2	0.806
Post-procedure thrombolysis in myocardial infarction (TIMI) flow				
0	5 (3.3%)	2 (2.1%)	3 (5.6%)	0.023
I	2 (1.3%)	2 (2.1%)	0 (0%)	
II	13 (8.6%)	4 (4.1%)	9 (16.7%)	
III	131 (86.8%)	89 (91.8%)	42 (77.8%)	
Re-intubation	7 (4.6%)	3 (3.1%)	4 (7.4%)	0.227
Days in intensive care unit				
1	53 (35.1%)	29 (29.9%)	24 (44.4%)	0.017
2	58 (38.4%)	45 (46.4%)	13 (24.1%)	
3	32 (21.2%)	20 (20.6%)	12 (22.2%)	
4	5 (3.3%)	3 (3.1%)	2 (3.7%)	
5	3 (2%)	0 (0%)	3 (5.6%)	
Bleeding	10 (6.6%)	7 (7.2%)	3 (5.6%)	0.694
Need of renal replacement therapy	5 (3.3%)	2 (2.1%)	3 (5.6%)	0.250
Re-infarction	8 (5.3%)	4 (4.1%)	4 (7.4%)	0.388
Mortality	42 (27.8%)	8 (8.2%)	34 (63%)	<0.001
Arrhythmias	38 (25.2%)	19 (19.6%)	19 (35.2%)	0.034
Asystole	1 (2.6%)	1 (5.3%)	0 (0%)	0.311
Pulseless electrical activity	4 (10.5%)	2 (10.5%)	2 (10.5%)	>0.999
Ventricular tachycardia	25 (65.8%)	14 (73.7%)	11 (57.9%)	0.305
Ventricular fibrillation	2 (5.3%)	1 (5.3%)	1 (5.3%)	>0.999
Bradyarrest	7 (18.4%)	3 (15.8%)	4 (21.1%)	0.676
AV block	2 (5.3%)	1 (5.3%)	1 (5.3%)	>0.999
Vasopressors/Inotropic support	121 (80.1%)	69 (71.1%)	52 (96.3%)	<0.001
Norepinephrine	114 (94.2%)	64 (92.8%)	50 (96.2%)	0.428
Dobutamine	12 (9.9%)	5 (7.2%)	7 (13.5%)	0.258
Levophed	5 (4.1%)	4 (5.8%)	1 (1.9%)	0.289
Epinephrine	4 (3.3%)	2 (2.9%)	2 (3.8%)	0.773
Inter-aortic balloon pump (IABP)	31 (20.5%)	20 (20.6%)	11 (20.4%)	0.987
Number of days on IABP	1.8 ± 0.9	1.9 ± 1	1.5 ± 0.5	0.222
Length of hospital stay (days)	2.1 ± 1.2	2.1 ± 1.1	2.2 ± 1.4	0.733

**Figure 1:**
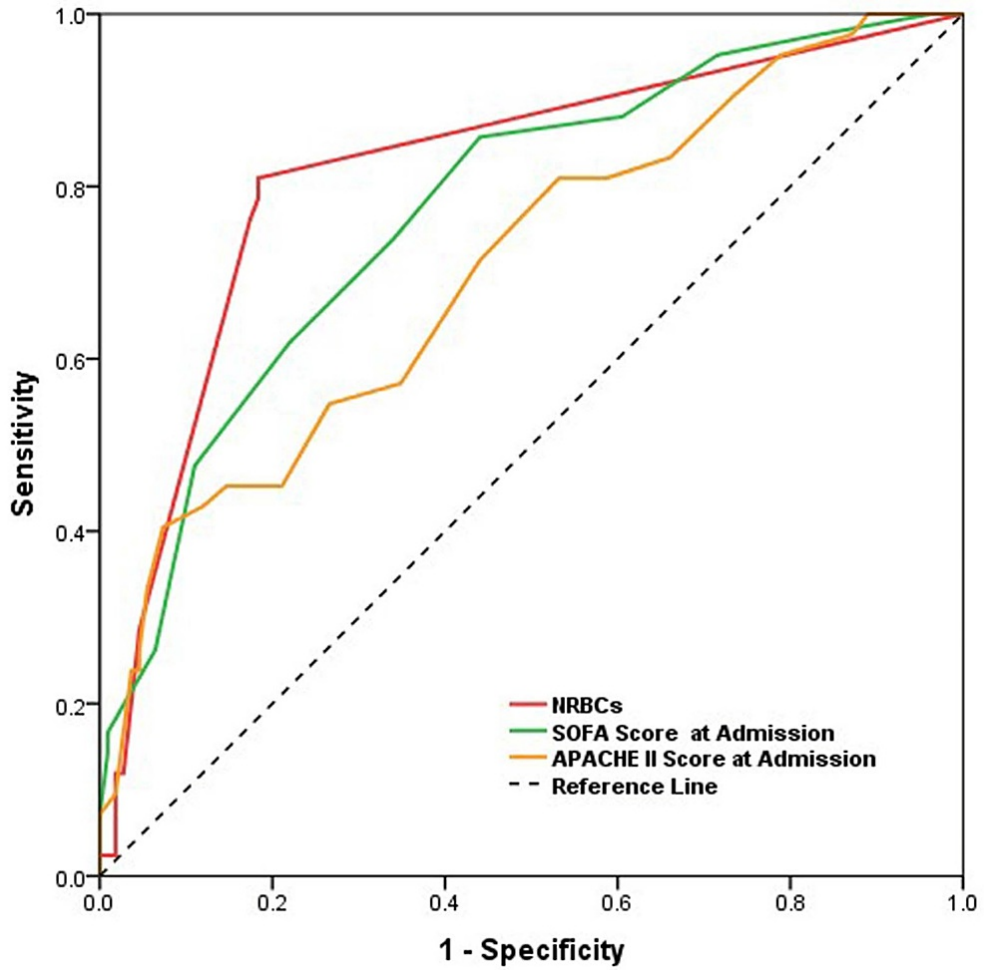
The receiver operating characteristic curve analysis for NRBCs, the APACHE II score, and the SOFA score for discrimination of ICU mortality NRBCs: Nucleated red blood cells; SOFA: Sequential organ failure assessment; APACHE: Acute physiology and chronic health evaluation.

The NRBCs were found to have significantly better discrimination power than the SOFA and APACHE II scores, with the AUC of 0.818 (95% CI: 0.738-0.899) vs. 0.774 (95% CI: 0.692-0.857) vs. 0.707 (95% CI: 0.613-0.801), respectively (Figure 1). The positive NRBCs have a sensitivity of 81.0% and a specificity of 81.7% for detecting ICU mortality.

## Discussion

The NRBCs are typically immature forms of RBCs that still contain a nucleus. In a healthy adult, RBCs normally lose their nuclei during maturation in the bone marrow and enter circulation as non-nucleated cells. The presence of NRBCs in peripheral blood is considered abnormal and can have various clinical implications [9]. Therefore, we conducted this study to evaluate the prognostic role of positive NRBCs and compared it with widely employed SOFA and APACHE II scores. The findings from this study provide valuable insights into the prognostic significance of positive NRBCs in patients with STEMI. In this cohort of 151 patients, identifying positive NRBCs in 35.8% of patients holds critical clinical implications. The association of positive NRBCs with higher stenosis (%) in the culprit vessel (98.8 ± 3.0% vs. 96.8 ± 5.7%; p = 0.004) underscores the potential relationship between this hematological marker and the extent of coronary artery disease. Furthermore, the correlation between positive NRBCs and impaired post-procedure TIMI flow (p = 0.023) suggests a potential link between NRBCs and impaired microvascular perfusion, which could contribute to worse outcomes. The study's most significant findings revolve around the association between positive NRBCs and adverse clinical outcomes. Patients with positive NRBCs displayed substantially higher rates of ICU mortality (63% vs. 8.2%; p < 0.001), arrhythmias (35.2% vs. 19.6%; p = 0.034), and an increased requirement for vasopressors/inotropic support (96.3% vs. 71.1%; p < 0.001) compared to those with negative NRBCs. These associations emphasize the potential role of positive NRBCs as a powerful predictor of patient outcomes, capturing both mortality and morbidity risks.

Furthermore, assessing the prognostic value of positive NRBCs compared to widely used scoring systems revealed promising results. Positive NRBCs demonstrated superior discrimination power compared to the SOFA and APACHE II scores. The greater AUC for positive NRBCs (0.818) signifies its ability to more accurately predict ICU mortality, as supported by its sensitivity of 81.0% and specificity of 81.7%. In line with our findings, a study conducted by Monteiro Júnior JG et al. [21] revealed an association between the presence of NRBCs in peripheral blood in patients with acute myocardial infarction (AMI) and mortality (hazard ratio (HR) 2.42, 95% CI: 1.35-4.36, p = 0.003). Similarly, Stachon A et al. [22] investigated this parameter in the ICU context. They concluded that NRBC presence could serve as a daily indicator of high mortality risk, suggesting against relocating NRBC-positive patients to a standard ward. Desai S et al. [23] established a link between NRBC positivity and higher mortality rates in patients with surgical sepsis (27% vs. 12%, p = 0.007). Additionally, Kuert S et al. [24] associated NRBCs with arterial oxygen partial tension, noting lower pO2 levels in patients who eventually died than survivors before NRBC detection. The potential mechanisms behind increased mortality in patients with elevated NRBCs encompass hypoxemia and systemic inflammation [22-25]. These hematological parameters directly correlate with the severity of systemic inflammation and hypoxemia, both pivotal factors in organic dysfunction pathophysiology. Early cardiac ischemic injuries trigger a robust inflammatory response, and subsequent conditions such as sepsis and shock may emerge during hospitalization [26]. Intriguingly, our study reported a notably higher NRBC prevalence (35.8%) than prior research, where frequencies ranged from 17.5% to 28.6% [22-25].

While information regarding the prognostic role of NRBCs in the AMI setting remains limited, Júnior JG et al. [26] proposed a hematological scoring system based on NRBCs, mean platelet volume (MPV), and neutrophil-to-lymphocyte ratio (NLR). Their study demonstrated that the presence of NRBCs (odds ratio: 33.9 (15.8 to 72.8)), increases in MPV (odds ratio 3.32 (1.46 to 7.55)), and NLR (odds ratio: 16.0 (5.67 to 45.0)) in peripheral blood were linked to poorer prognosis. At a cut-off of 26 points, the scoring system exhibited a sensitivity of 89.1%, specificity of 67.2%, negative predictive value of 97.9%, and positive predictive value of 26.8% [26]. Over the past five decades, scientific advancements and the introduction of automated peripheral blood cell counting have transformed complete blood cell counts into vital clinical tools for identifying hematopoietic responses to existing injury [26,27]. Consequently, these variables not only mirror ischemia and its hemodynamic repercussions but also encapsulate inflammatory processes, infectious or not-during hospitalization, that might contribute to higher AMI patient mortality rates. These insights complement clinical practice's current risk stratification scores [26]. Nevertheless, it is important to acknowledge several key limitations of our study. These encompass a small sample size and a single-center design, potentially limiting the generalizability of our findings to a broader population. Conducting the study within a solitary center may introduce biases related to unique patient characteristics and healthcare practices within that specific institution. To enhance the applicability and credibility of our findings, future studies should involve multiple centers and diverse patient populations to validate and extend our conclusions.

## Conclusions

NRBCs in the peripheral blood of patients presenting with STEMI have a significant linear relation to mortality. Furthermore, the current investigation highlights the potential superiority of positive NRBCs when compared to widely employed scoring systems such as the SOFA and APACHE II. Thus, including NRBC monitoring for prognostic evaluation and stratification of in-hospital mortality can aid clinical decision-making, ultimately optimizing patient care strategies for those with STEMI.
